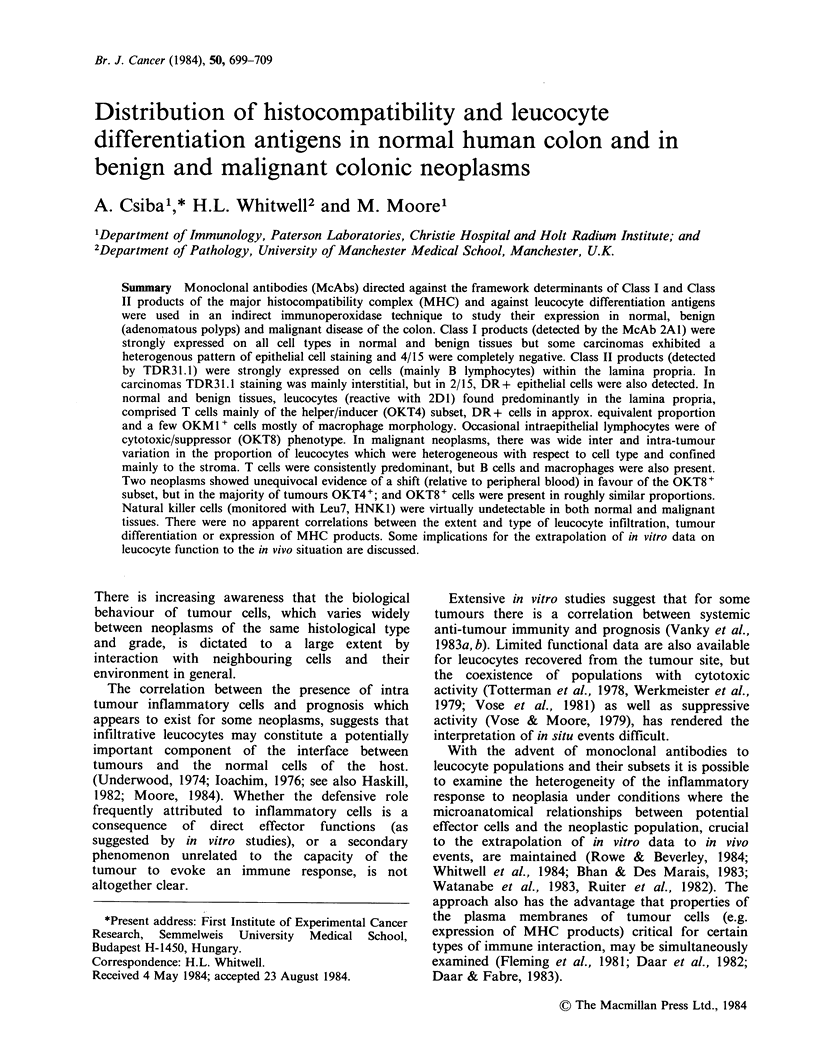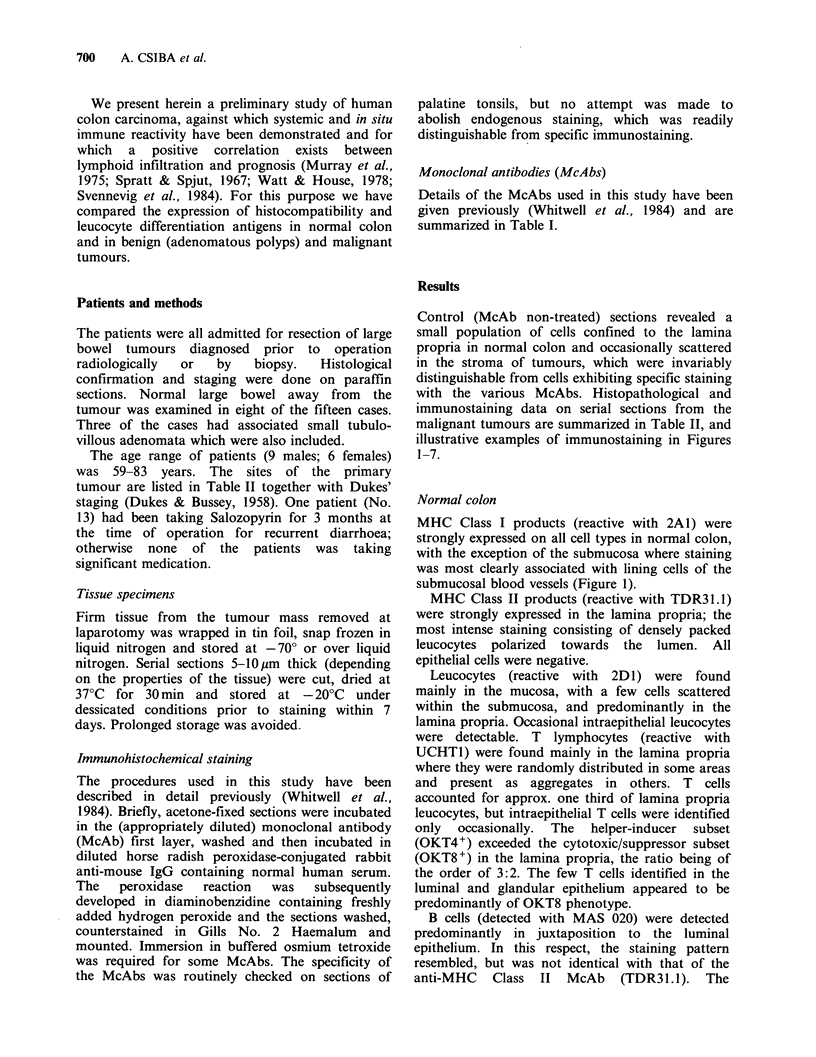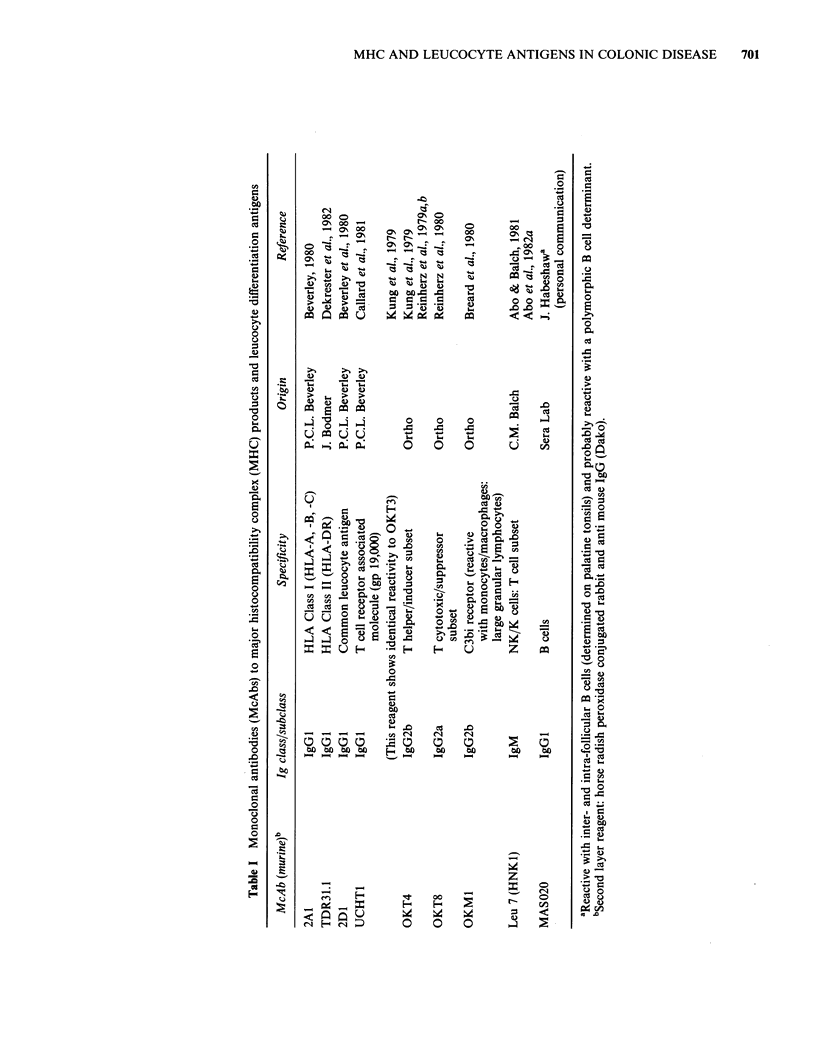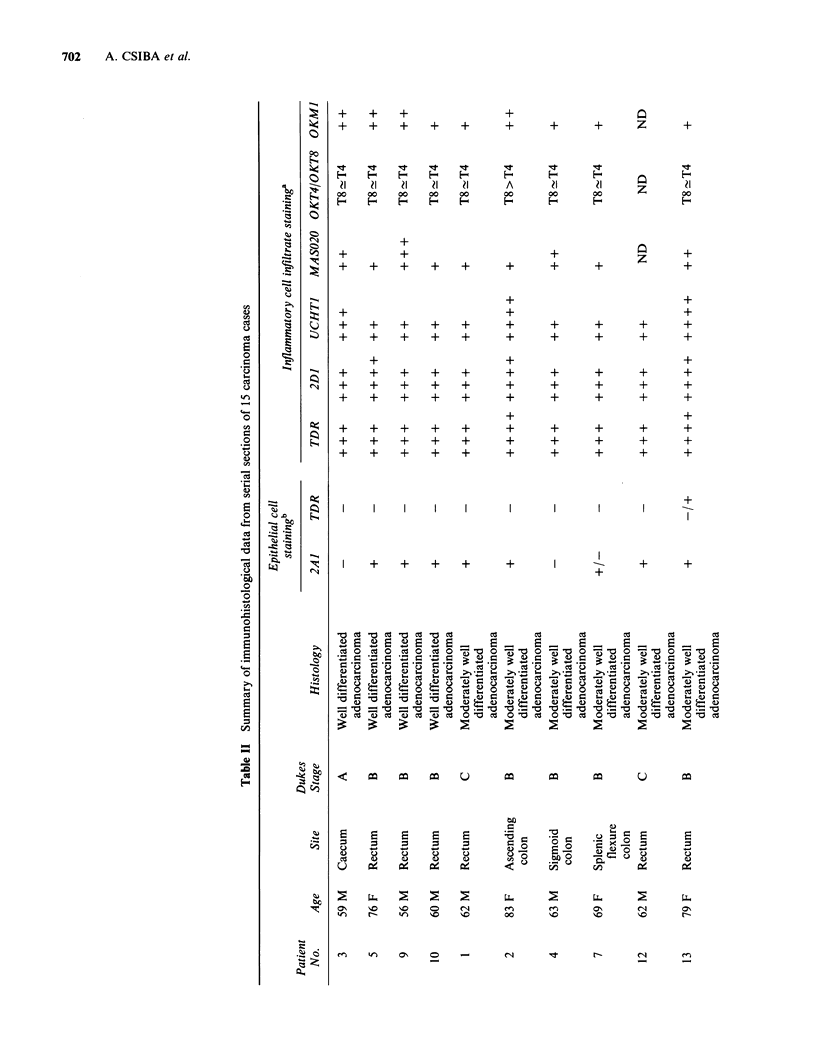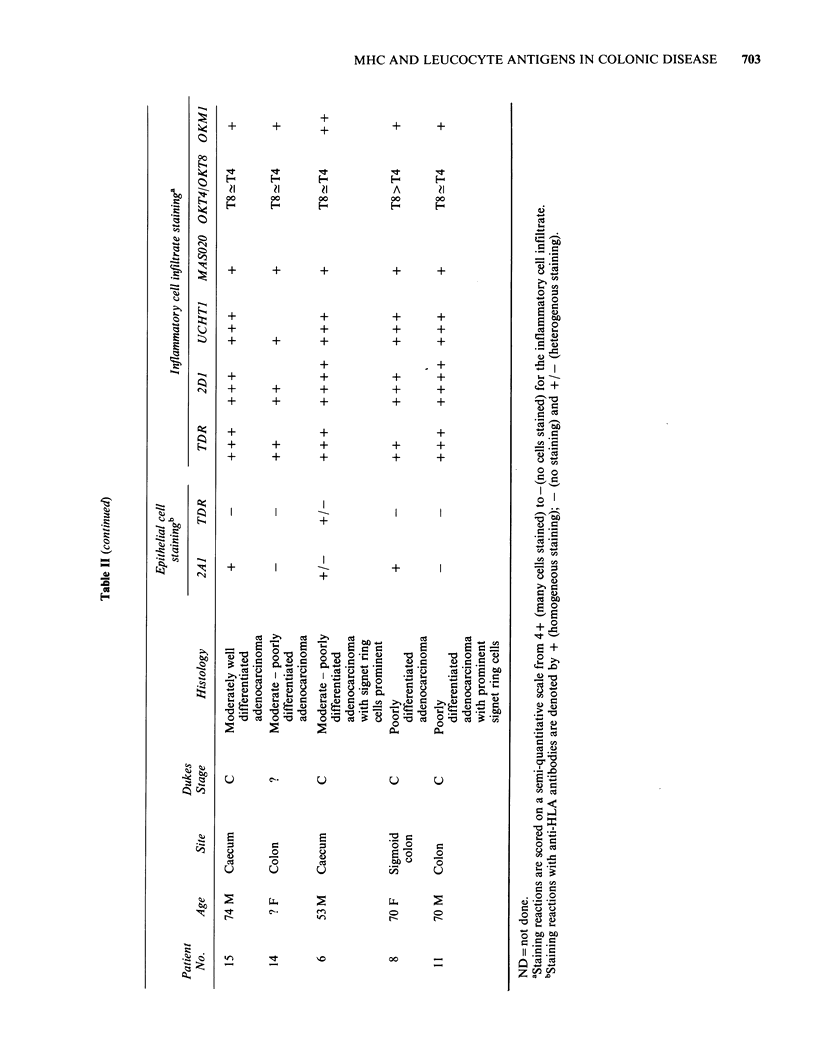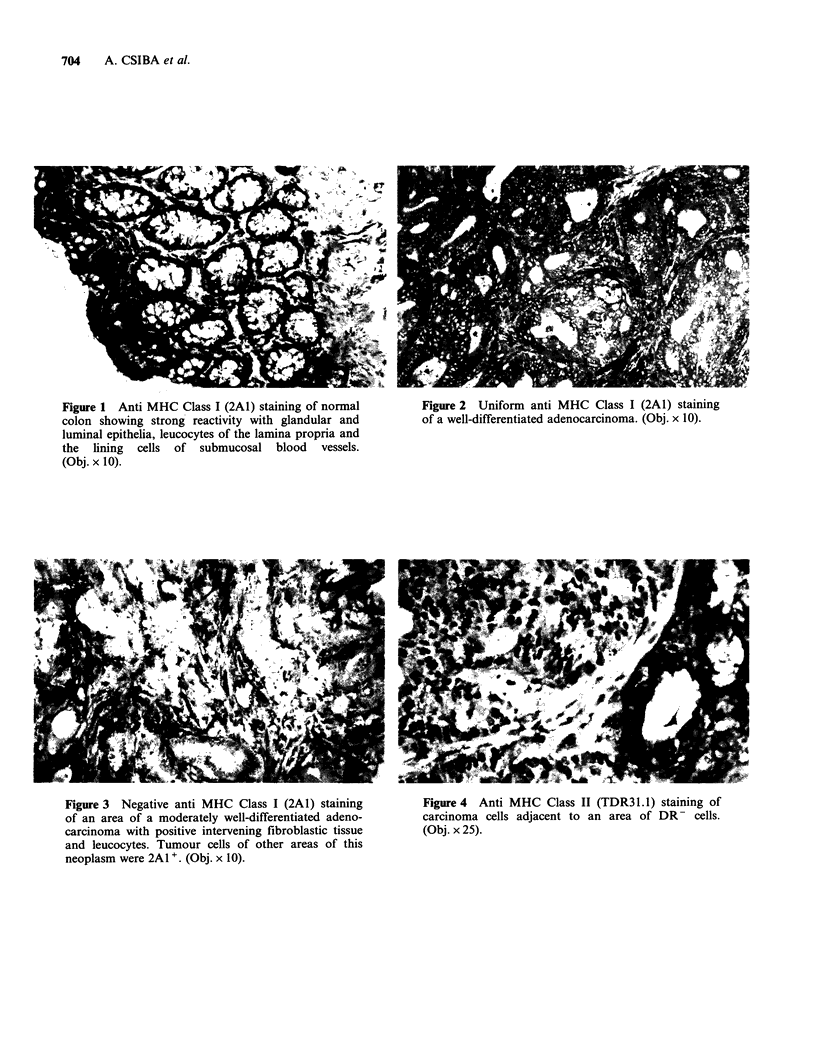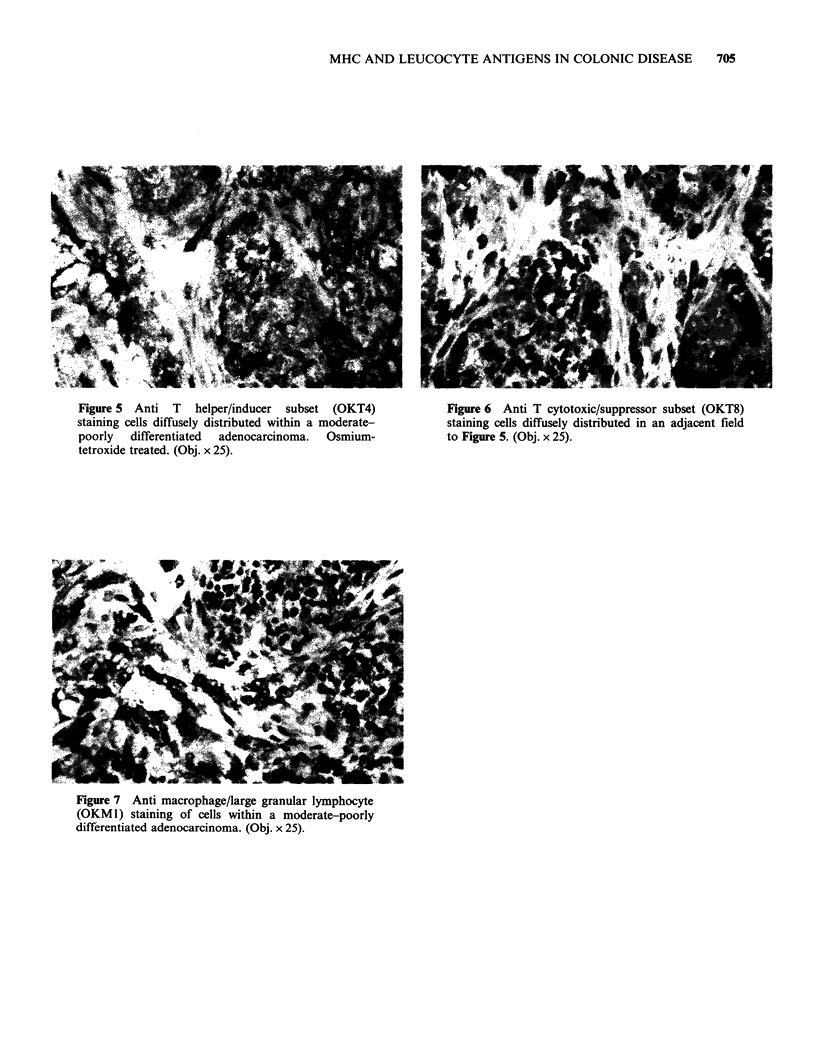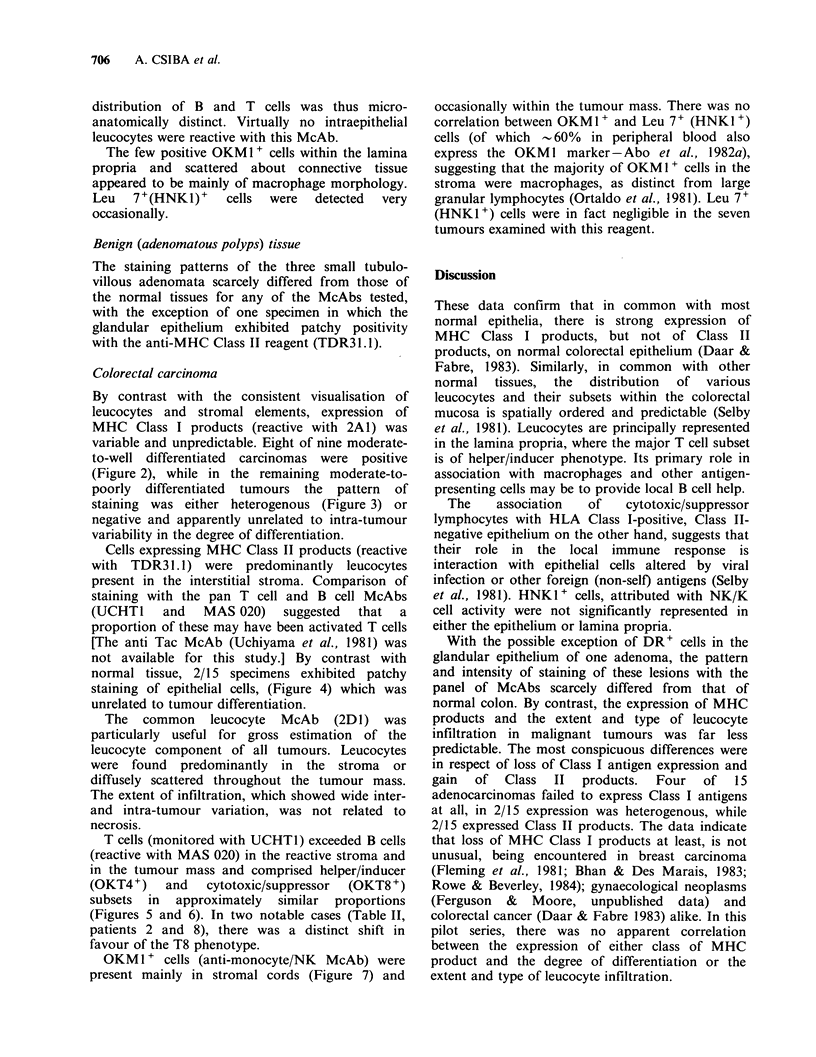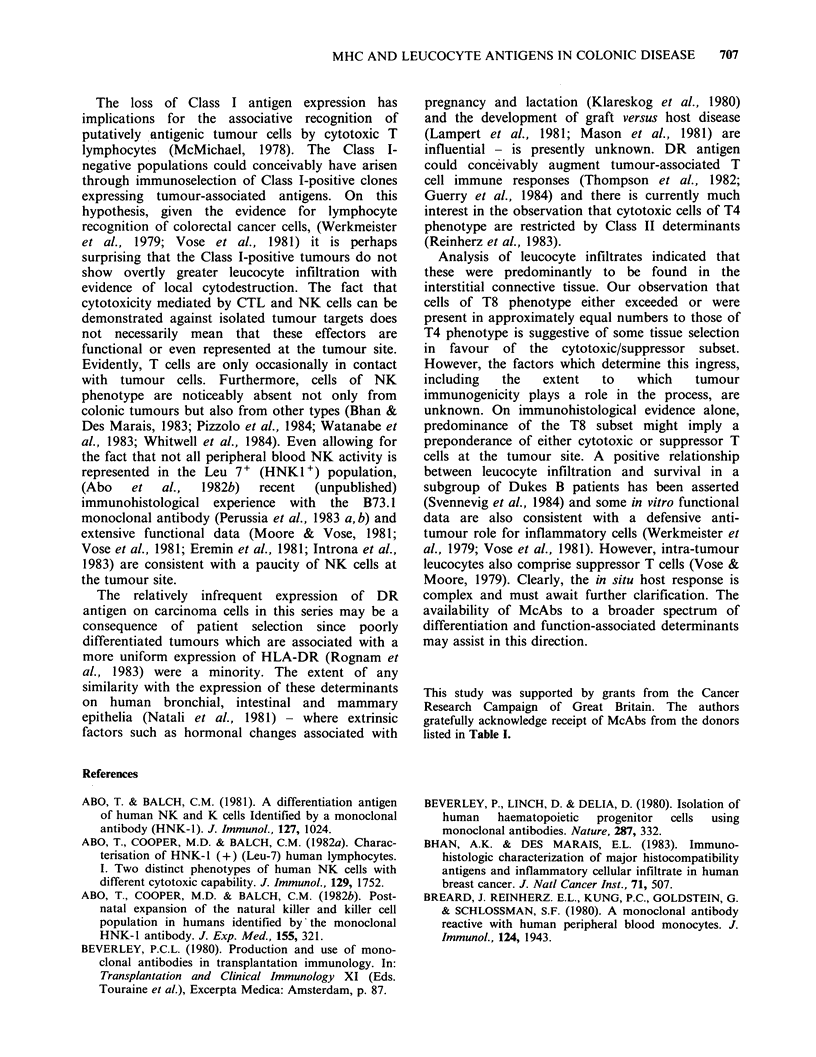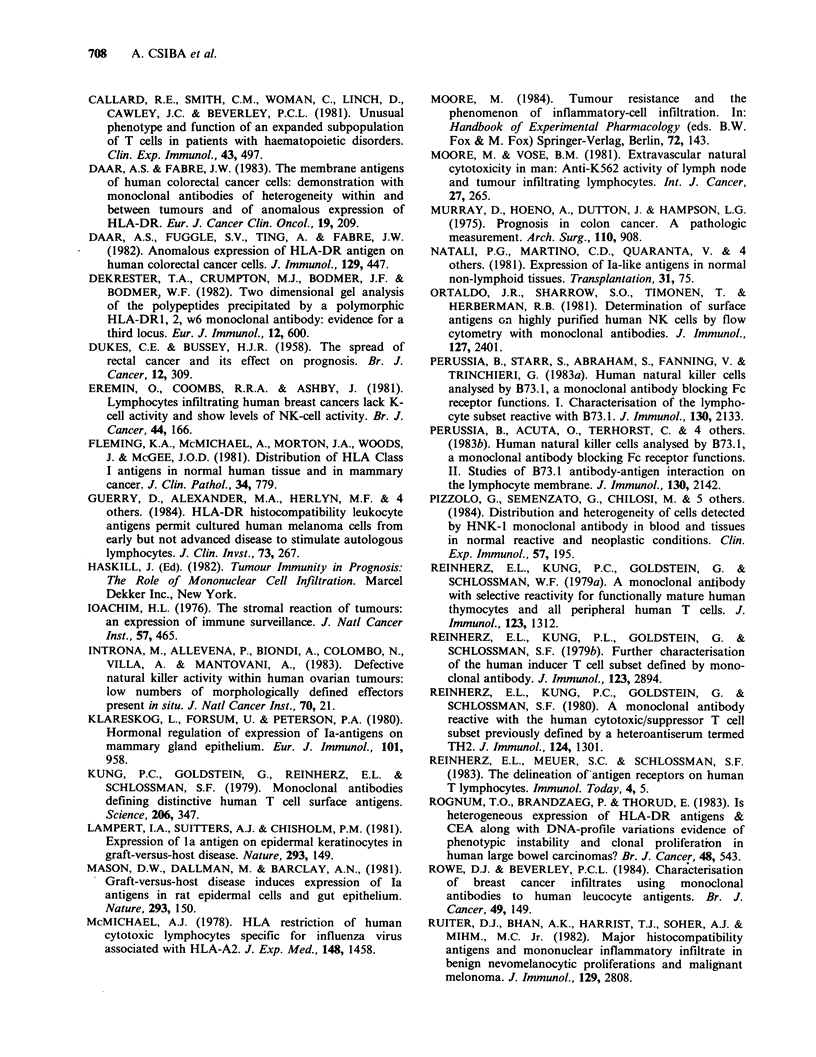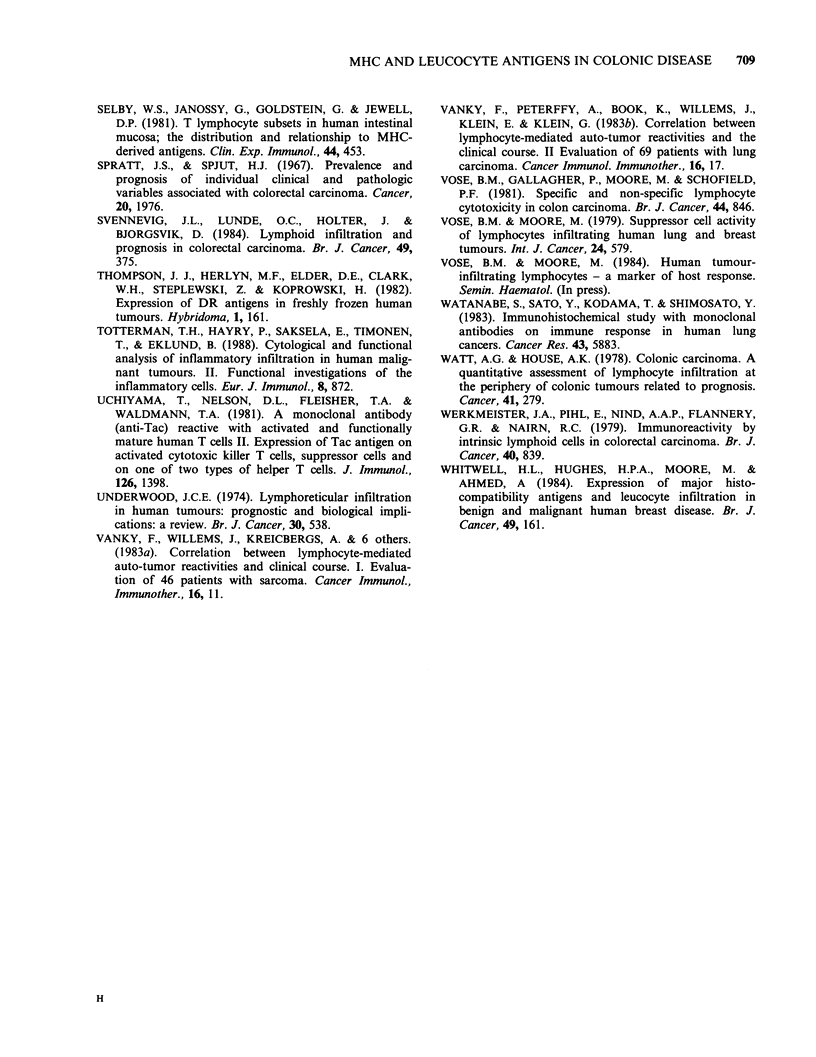# Distribution of histocompatibility and leucocyte differentiation antigens in normal human colon and in benign and malignant colonic neoplasms.

**DOI:** 10.1038/bjc.1984.239

**Published:** 1984-11

**Authors:** A. Csiba, H. L. Whitwell, M. Moore

## Abstract

**Images:**


*Br. J. Cancer* (1984), **50**, 699–709

### Distribution of histocompatibility and leucocyte differentiation antigens in normal human colon and in benign and malignant colonic neoplasms

A. Csiba[1],* H.L. Whitwell[2] and M. Moore[1]

[1]*Department of Immunology, Paterson Laboratories, Christie Hospital and Holt Radium Institute; and* [2]*Department of Pathology, University of Manchester Medical School, Manchester, U.K.*

**Summary** Monoclonal antibodies (McAbs) directed against the framework determinants of Class I and Class II products of the major histocompatibility complex (MHC) and against leucocyte differentiation antigens were used in an indirect immunoperoxidase technique to study their expression in normal, benign (adenomatous polyps) and malignant disease of the colon. Class I products (detected by the McAb 2A1) were strongly expressed on all cell types in normal and benign tissues but some carcinomas exhibited a heterogenous pattern of epithelial cell staining and 4/15 were completely negative. Class II products (detected by TDR31.1) were strongly expressed on cells (mainly B lymphocytes) within the lamina propria. In carcinomas TDR31.1 staining was mainly interstitial, but in 2/15, DR+ epithelial cells were also detected. In normal and benign tissues, leucocytes (reactive with 2D1) found predominantly in the lamina propria, comprised T cells mainly of the helper/inducer (OKT4) subset, DR+ cells in approx. equivalent proportion and a few OKM1$^+$ cells mostly of macrophage morphology. Occasional intraepithelial lymphocytes were of cytotoxic/suppressor (OKT8) phenotype. In malignant neoplasms, there was wide inter and intra-tumour variation in the proportion of leucocytes which were heterogeneous with respect to cell type and confined mainly to the stroma. T cells were consistently predominant, but B cells and macrophages were also present. Two neoplasms showed unequivocal evidence of a shift (relative to peripheral blood) in favour of the OKT8$^+$ subset, but in the majority of tumours OKT4$^+$; and OKT8$^+$ cells were present in roughly similar proportions. Natural killer cells (monitored with Leu7, HNK1) were virtually undetectable in both normal and malignant tissues. There were no apparent correlations between the extent and type of leucocyte infiltration, tumour differentiation or expression of MHC products. Some implications for the extrapolation of *in vitro* data on leucocyte function to the *in vivo* situation are discussed.

There is increasing awareness that the biological behaviour of tumour cells, which varies widely between neoplasms of the same histological type and grade, is dictated to a large extent by interaction with neighbouring cells and their environment in general.

The correlation between the presence of intra tumour inflammatory cells and prognosis which appears to exist for some neoplasms, suggests that infiltrative leucocytes may constitute a potentially important component of the interface between tumours and the normal cells of the host. (Underwood, 1974; Ioachim, 1976; see also Haskill, 1982; Moore, 1984). Whether the defensive role frequently attributed to inflammatory cells is a consequence of direct effector functions (as suggested by *in vitro* studies), or a secondary phenomenon unrelated to the capacity of the tumour to evoke an immune response, is not altogether clear.

*Present address: First Institute of Experimental Cancer Research, Semmelweis University Medical School, Budapest H-1450, Hungary.
Correspondence: H.L. Whitwell.
Received 4 May 1984; accepted 23 August 1984.

Extensive *in vitro* studies suggest that for some tumours there is a correlation between systemic anti-tumour immunity and prognosis (Vanky *et al.*, 1983*a, b*). Limited functional data are also available for leucocytes recovered from the tumour site, but the coexistence of populations with cytotoxic activity (Totterman *et al.*, 1978, Werkmeister *et al.*, 1979; Vose *et al.*, 1981) as well as suppressive activity (Vose & Moore, 1979), has rendered the interpretation of *in situ* events difficult.

With the advent of monoclonal antibodies to leucocyte populations and their subsets it is possible to examine the heterogeneity of the inflammatory response to neoplasia under conditions where the microanatomical relationships between potential effector cells and the neoplastic population, crucial to the extrapolation of *in vitro* data to *in vivo* events, are maintained (Rowe & Beverley, 1984; Whitwell *et al.*, 1984; Bhan & Des Marais, 1983; Watanabe *et al.*, 1983, Ruiter *et al.*, 1982). The approach also has the advantage that properties of the plasma membranes of tumour cells (e.g. expression of MHC products) critical for certain types of immune interaction, may be simultaneously examined (Fleming *et al.*, 1981; Daar *et al.*, 1982; Daar & Fabre, 1983).

© The Macmillan Press Ltd., 1984

We present herein a preliminary study of human colon carcinoma, against which systemic and *in situ* immune reactivity have been demonstrated and for which a positive correlation exists between lymphoid infiltration and prognosis (Murray *et al.*, 1975; Spratt & Spjut, 1967; Watt & House, 1978; Svennevig *et al.*, 1984). For this purpose we have compared the expression of histocompatibility and leucocyte differentiation antigens in normal colon and in benign (adenomatous polyps) and malignant tumours.

### Patients and methods

The patients were all admitted for resection of large bowel tumours diagnosed prior to operation radiologically or by biopsy. Histological confirmation and staging were done on paraffin sections. Normal large bowel away from the tumour was examined in eight of the fifteen cases. Three of the cases had associated small tubulovillous adenomata which were also included.

The age range of patients (9 males; 6 females) was 59–83 years. The sites of the primary tumour are listed in Table II together with Dukes' staging (Dukes & Bussey, 1958). One patient (No. 13) had been taking Salozopyrin for 3 months at the time of operation for recurrent diarrhoea; otherwise none of the patients was taking significant medication.

### *Tissue specimens*

Firm tissue from the tumour mass removed at laparotomy was wrapped in tin foil, snap frozen in liquid nitrogen and stored at −70° or over liquid nitrogen. Serial sections 5–10 μm thick (depending on the properties of the tissue) were cut, dried at 37°C for 30 min and stored at −20°C under dessicated conditions prior to staining within 7 days. Prolonged storage was avoided.

### *Immunohistochemical staining*

The procedures used in this study have been described in detail previously (Whitwell *et al.*, 1984). Briefly, acetone-fixed sections were incubated in the (appropriately diluted) monoclonal antibody (McAb) first layer, washed and then incubated in diluted horse radish peroxidase-conjugated rabbit anti-mouse IgG containing normal human serum. The peroxidase reaction was subsequently developed in diaminobenzidine containing freshly added hydrogen peroxide and the sections washed, counterstained in Gills No. 2 Haemalum and mounted. Immersion in buffered osmium tetroxide was required for some McAbs. The specificity of the McAbs was routinely checked on sections of palatine tonsils, but no attempt was made to abolish endogenous staining, which was readily distinguishable from specific immunostaining.

### *Monoclonal antibodies (McAbs)*

Details of the McAbs used in this study have been given previously (Whitwell *et al.*, 1984) and are summarized in Table I.

### Results

Control (McAb non-treated) sections revealed a small population of cells confined to the lamina propria in normal colon and occasionally scattered in the stroma of tumours, which were invariably distinguishable from cells exhibiting specific staining with the various McAbs. Histopathological and immunostaining data on serial sections from the malignant tumours are summarized in Table II, and illustrative examples of immunostaining in Figures 1–7.

### *Normal colon*

MHC Class I products (reactive with 2A1) were strongly expressed on all cell types in normal colon, with the exception of the submucosa where staining was most clearly associated with lining cells of the submucosal blood vessels (Figure 1).

MHC Class II products (reactive with TDR31.1) were strongly expressed in the lamina propria; the most intense staining consisting of densely packed leucocytes polarized towards the lumen. All epithelial cells were negative.

Leucocytes (reactive with 2D1) were found mainly in the mucosa, with a few cells scattered within the submucosa, and predominantly in the lamina propria. Occasional intraepithelial leucocytes were detectable. T lymphocytes (reactive with UCHT1) were found mainly in the lamina propria where they were randomly distributed in some areas and present as aggregates in others. T cells accounted for approx. one third of lamina propria leucocytes, but intraepithelial T cells were identified only occasionally. The helper-inducer subset ($OKT4^+$) exceeded the cytotoxic/suppressor subset ($OKT8^+$) in the lamina propria, the ratio being of the order of 3:2. The few T cells identified in the luminal and glandular epithelium appeared to be predominantly of OKT8 phenotype.

B cells (detected with MAS 020) were detected predominantly in juxtaposition to the luminal epithelium. In this respect, the staining pattern resembled, but was not identical with that of the anti-MHC Class II McAb (TDR31.1). The

**Table I** Monoclonal antibodies (McAbs) to major histocompatibility complex (MHC) products and leucocyte differentiation antigens

| *McAb (murine)*[b] | *Ig class/subclass* | *Specificity* | *Origin* | *Reference* |
|---|---|---|---|---|
| 2A1 | IgG1 | HLA Class I (HLA-A, -B, -C) | P.C.L. Beverley | Beverley, 1980 |
| TDR31.1 | IgG1 | HLA Class II (HLA-DR) | J. Bodmer | Dekrester *et al.*, 1982 |
| 2D1 | IgG1 | Common leucocyte antigen | P.C.L. Beverley | Beverley *et al.*, 1980 |
| UCHT1 | IgG1 | T cell receptor associated molecule (gp 19,000) | P.C.L. Beverley | Callard *et al.*, 1981 |
| | (This reagent shows identical reactivity to OKT3) | | | Kung *et al.*, 1979 |
| OKT4 | IgG2b | T helper/inducer subset | Ortho | Kung *et al.*, 1979<br>Reinherz *et al.*, 1979*a,b* |
| OKT8 | IgG2a | T cytotoxic/suppressor subset | Ortho | Reinherz *et al.*, 1980 |
| OKM1 | IgG2b | C3bi receptor (reactive with monocytes/macrophages: large granular lymphocytes) | Ortho | Breard *et al.*, 1980 |
| Leu 7 (HNK1) | IgM | NK/K cells: T cell subset | C.M. Balch | Abo & Balch, 1981<br>Abo *et al.*, 1982*a* |
| MAS020 | IgG1 | B cells | Sera Lab | J. Habeshaw[a] (personal communication) |

[a]Reactive with inter- and intra-follicular B cells (determined on palatine tonsils) and probably reactive with a polymorphic B cell determinant.

[b]Second layer reagent: horse radish peroxidase conjugated rabbit and anti mouse IgG (Dako).

**Table II** Summary of immunohistological data from serial sections of 15 carcinoma cases

| Patient No. | Age | Site | Dukes Stage | Histology | *Epithelial cell staining*[b] | | *Inflammatory cell infiltrate staining*[a] | | | | | |
|---|---|---|---|---|---|---|---|---|---|---|---|---|
| | | | | | *2A1* | *TDR* | *TDR* | *2D1* | *UCHT1* | *MAS020* | *OKT4/OKT8* | *OKM1* |
| 3 | 59 M | Caecum | A | Well differentiated adenocarcinoma | − | − | +++ | +++ | +++ | ++ | T8≃T4 | ++ |
| 5 | 76 F | Rectum | B | Well differentiated adenocarcinoma | + | − | +++ | ++++ | ++ | + | T8≃T4 | ++ |
| 9 | 56 M | Rectum | B | Well differentiated adenocarcinoma | + | − | +++ | +++ | ++ | +++ | T8≃T4 | ++ |
| 10 | 60 M | Rectum | B | Well differentiated adenocarcinoma | + | − | +++ | +++ | ++ | + | T8≃T4 | + |
| 1 | 62 M | Rectum | C | Moderately well differentiated adenocarcinoma | + | − | +++ | +++ | ++ | + | T8≃T4 | + |
| 2 | 83 F | Ascending colon | B | Moderately well differentiated adenocarcinoma | + | − | ++++ | ++++ | ++++ | + | T8>T4 | ++ |
| 4 | 63 M | Sigmoid colon | B | Moderately well differentiated adenocarcinoma | − | − | +++ | +++ | ++ | ++ | T8≃T4 | + |
| 7 | 69 F | Splenic flexure colon | B | Moderately well differentiated adenocarcinoma | +/− | − | +++ | +++ | ++ | + | T8≃T4 | + |
| 12 | 62 M | Rectum | C | Moderately well differentiated adenocarcinoma | + | − | +++ | +++ | ++ | ND | ND | ND |
| 13 | 79 F | Rectum | B | Moderately well differentiated adenocarcinoma | + | −/+ | ++++ | ++++ | ++++ | ++ | T8≃T4 | + |

**Table II** (*continued*)

| *Patient No.* | *Age* | *Site* | *Dukes Stage* | *Histology* | *Epithelial cell staining*[b] *2A1* | *Epithelial cell staining*[b] *TDR* | *Inflammatory cell infiltrate staining*[a] *TDR* | *2D1* | *UCHT1* | *MAS020* | *OKT4/OKT8* | *OKM1* |
|---|---|---|---|---|---|---|---|---|---|---|---|---|
| 15 | 74 M | Caecum | C | Moderately well differentiated adenocarcinoma | + | − | +++ | +++ | +++ | + | T8≃T4 | + |
| 14 | ? F | Colon | ? | Moderate – poorly differentiated adenocarcinoma | − | − | ++ | ++ | + | + | T8≃T4 | + |
| 6 | 53 M | Caecum | C | Moderate – poorly differentiated adenocarcinoma with signet ring cells prominent | +/− | +/− | +++ | ++++ | +++ | + | T8≃T4 | ++ |
| 8 | 70 F | Sigmoid colon | C | Poorly differentiated adenocarcinoma | + | − | ++ | +++ | +++ | + | T8>T4 | + |
| 11 | 70 M | Colon | C | Poorly differentiated adenocarcinoma with prominent signet ring cells | − | − | +++ | ++++ | +++ | + | T8≃T4 | + |

ND = not done.

[a]Staining reactions are scored on a semi-quantitative scale from 4+ (many cells stained) to − (no cells stained) for the inflammatory cell infiltrate.

[b]Staining reactions with anti-HLA antibodies are denoted by + (homogeneous staining); − (no staining) and +/− (heterogenous staining).

**Figure 1** Anti MHC Class I (2A1) staining of normal colon showing strong reactivity with glandular and luminal epithelia, leucocytes of the lamina propria and the lining cells of submucosal blood vessels. (Obj. × 10).

**Figure 2** Uniform anti MHC Class I (2A1) staining of a well-differentiated adenocarcinoma. (Obj. × 10).

**Figure 3** Negative anti MHC Class I (2A1) staining of an area of a moderately well-differentiated adenocarcinoma with positive intervening fibroblastic tissue and leucocytes. Tumour cells of other areas of this neoplasm were 2A1$^+$. (Obj. × 10).

**Figure 4** Anti MHC Class II (TDR31.1) staining of carcinoma cells adjacent to an area of DR$^-$ cells. (Obj. × 25).

**Figure 5** Anti T helper/inducer subset (OKT4) staining cells diffusely distributed within a moderate–poorly differentiated adenocarcinoma. Osmium-tetroxide treated. (Obj. × 25).

**Figure 6** Anti T cytotoxic/suppressor subset (OKT8) staining cells diffusely distributed in an adjacent field to **Figure 5**. (Obj. × 25).

**Figure 7** Anti macrophage/large granular lymphocyte (OKM1) staining of cells within a moderate–poorly differentiated adenocarcinoma. (Obj. × 25).

distribution of B and T cells was thus micro-anatomically distinct. Virtually no intraepithelial leucocytes were reactive with this McAb.

The few positive $OKM1^+$ cells within the lamina propria and scattered about connective tissue appeared to be mainly of macrophage morphology. Leu $7^+(HNK1)^+$ cells were detected very occasionally.

*Benign (adenomatous polyps) tissue*

The staining patterns of the three small tubulovillous adenomata scarcely differed from those of the normal tissues for any of the McAbs tested, with the exception of one specimen in which the glandular epithelium exhibited patchy positivity with the anti-MHC Class II reagent (TDR31.1).

*Colorectal carcinoma*

By contrast with the consistent visualisation of leucocytes and stromal elements, expression of MHC Class I products (reactive with 2A1) was variable and unpredictable. Eight of nine moderate-to-well differentiated carcinomas were positive (Figure 2), while in the remaining moderate-to-poorly differentiated tumours the pattern of staining was either heterogenous (Figure 3) or negative and apparently unrelated to intra-tumour variability in the degree of differentiation.

Cells expressing MHC Class II products (reactive with TDR31.1) were predominantly leucocytes present in the interstitial stroma. Comparison of staining with the pan T cell and B cell McAbs (UCHT1 and MAS 020) suggested that a proportion of these may have been activated T cells [The anti Tac McAb (Uchiyama *et al.*, 1981) was not available for this study.] By contrast with normal tissue, 2/15 specimens exhibited patchy staining of epithelial cells, (Figure 4) which was unrelated to tumour differentiation.

The common leucocyte McAb (2D1) was particularly useful for gross estimation of the leucocyte component of all tumours. Leucocytes were found predominantly in the stroma or diffusely scattered throughout the tumour mass. The extent of infiltration, which showed wide inter- and intra-tumour variation, was not related to necrosis.

T cells (monitored with UCHT1) exceeded B cells (reactive with MAS 020) in the reactive stroma and in the tumour mass and comprised helper/inducer ($OKT4^+$) and cytotoxic/suppressor ($OKT8^+$) subsets in approximately similar proportions (Figures 5 and 6). In two notable cases (Table II, patients 2 and 8), there was a distinct shift in favour of the T8 phenotype.

$OKM1^+$ cells (anti-monocyte/NK McAb) were present mainly in stromal cords (Figure 7) and occasionally within the tumour mass. There was no correlation between $OKM1^+$ and Leu $7^+$ ($HNK1^+$) cells (of which ~60% in peripheral blood also express the OKM1 marker—Abo *et al.*, 1982*a*), suggesting that the majority of $OKM1^+$ cells in the stroma were macrophages, as distinct from large granular lymphocytes (Ortaldo *et al.*, 1981). Leu $7^+$ ($HNK1^+$) cells were in fact negligible in the seven tumours examined with this reagent.

### Discussion

These data confirm that in common with most normal epithelia, there is strong expression of MHC Class I products, but not of Class II products, on normal colorectal epithelium (Daar & Fabre, 1983). Similarly, in common with other normal tissues, the distribution of various leucocytes and their subsets within the colorectal mucosa is spatially ordered and predictable (Selby *et al.*, 1981). Leucocytes are principally represented in the lamina propria, where the major T cell subset is of helper/inducer phenotype. Its primary role in association with macrophages and other antigen-presenting cells may be to provide local B cell help.

The association of cytotoxic/suppressor lymphocytes with HLA Class I-positive, Class II-negative epithelium on the other hand, suggests that their role in the local immune response is interaction with epithelial cells altered by viral infection or other foreign (non-self) antigens (Selby *et al.*, 1981). $HNK1^+$ cells, attributed with NK/K cell activity were not significantly represented in either the epithelium or lamina propria.

With the possible exception of $DR^+$ cells in the glandular epithelium of one adenoma, the pattern and intensity of staining of these lesions with the panel of McAbs scarcely differed from that of normal colon. By contrast, the expression of MHC products and the extent and type of leucocyte infiltration in malignant tumours was far less predictable. The most conspicuous differences were in respect of loss of Class I antigen expression and gain of Class II products. Four of 15 adenocarcinomas failed to express Class I antigens at all, in 2/15 expression was heterogenous, while 2/15 expressed Class II products. The data indicate that loss of MHC Class I products at least, is not unusual, being encountered in breast carcinoma (Fleming *et al.*, 1981; Bhan & Des Marais, 1983; Rowe & Beverley, 1984); gynaecological neoplasms (Ferguson & Moore, unpublished data) and colorectal cancer (Daar & Fabre 1983) alike. In this pilot series, there was no apparent correlation between the expression of either class of MHC product and the degree of differentiation or the extent and type of leucocyte infiltration.

The loss of Class I antigen expression has implications for the associative recognition of putatively antigenic tumour cells by cytotoxic T lymphocytes (McMichael, 1978). The Class I-negative populations could conceivably have arisen through immunoselection of Class I-positive clones expressing tumour-associated antigens. On this hypothesis, given the evidence for lymphocyte recognition of colorectal cancer cells, (Werkmeister *et al.*, 1979; Vose *et al.*, 1981) it is perhaps surprising that the Class I-positive tumours do not show overtly greater leucocyte infiltration with evidence of local cytodestruction. The fact that cytotoxicity mediated by CTL and NK cells can be demonstrated against isolated tumour targets does not necessarily mean that these effectors are functional or even represented at the tumour site. Evidently, T cells are only occasionally in contact with tumour cells. Furthermore, cells of NK phenotype are noticeably absent not only from colonic tumours but also from other types (Bhan & Des Marais, 1983; Pizzolo *et al.*, 1984; Watanabe *et al.*, 1983; Whitwell *et al.*, 1984). Even allowing for the fact that not all peripheral blood NK activity is represented in the Leu $7^+$ ($HNK1^+$) population, (Abo *et al.*, 1982*b*) recent (unpublished) immunohistological experience with the B73.1 monoclonal antibody (Perussia *et al.*, 1983 *a,b*) and extensive functional data (Moore & Vose, 1981; Vose *et al.*, 1981; Eremin *et al.*, 1981; Introna *et al.*, 1983) are consistent with a paucity of NK cells at the tumour site.

The relatively infrequent expression of DR antigen on carcinoma cells in this series may be a consequence of patient selection since poorly differentiated tumours which are associated with a more uniform expression of HLA-DR (Rognam *et al.*, 1983) were a minority. The extent of any similarity with the expression of these determinants on human bronchial, intestinal and mammary epithelia (Natali *et al.*, 1981) – where extrinsic factors such as hormonal changes associated with pregnancy and lactation (Klareskog *et al.*, 1980) and the development of graft *versus* host disease (Lampert *et al.*, 1981; Mason *et al.*, 1981) are influential – is presently unknown. DR antigen could conceivably augment tumour-associated T cell immune responses (Thompson *et al.*, 1982; Guerry *et al.*, 1984) and there is currently much interest in the observation that cytotoxic cells of T4 phenotype are restricted by Class II determinants (Reinherz *et al.*, 1983).

Analysis of leucocyte infiltrates indicated that these were predominantly to be found in the interstitial connective tissue. Our observation that cells of T8 phenotype either exceeded or were present in approximately equal numbers to those of T4 phenotype is suggestive of some tissue selection in favour of the cytotoxic/suppressor subset. However, the factors which determine this ingress, including the extent to which tumour immunogenicity plays a role in the process, are unknown. On immunohistological evidence alone, predominance of the T8 subset might imply a preponderance of either cytotoxic or suppressor T cells at the tumour site. A positive relationship between leucocyte infiltration and survival in a subgroup of Dukes B patients has been asserted (Svennevig *et al.*, 1984) and some *in vitro* functional data are also consistent with a defensive anti-tumour role for inflammatory cells (Werkmeister *et al.*, 1979; Vose *et al.*, 1981). However, intra-tumour leucocytes also comprise suppressor T cells (Vose & Moore, 1979). Clearly, the *in situ* host response is complex and must await further clarification. The availability of McAbs to a broader spectrum of differentiation and function-associated determinants may assist in this direction.

This study was supported by grants from the Cancer Research Campaign of Great Britain. The authors gratefully acknowledge receipt of McAbs from the donors listed in **Table I.**

### References

ABO, T. & BALCH, C.M. (1981). A differentiation antigen of human NK and K cells Identified by a monoclonal antibody (HNK-1). *J. Immunol.*, **127**, 1024.

ABO, T., COOPER, M.D. & BALCH, C.M. (1982*a*). Characterisation of HNK-1 (+) (Leu-7) human lymphocytes. I. Two distinct phenotypes of human NK cells with different cytotoxic capability. *J. Immunol.*, **129**, 1752.

ABO, T., COOPER, M.D. & BALCH, C.M. (1982*b*). Post-natal expansion of the natural killer and killer cell population in humans identified by the monoclonal HNK-1 antibody. *J. Exp. Med.*, **155**, 321.

BEVERLEY, P.C.L. (1980). Production and use of monoclonal antibodies in transplantation immunology. In: *Transplantation and Clinical Immunology* XI (Eds. Touraine *et al.*), Excerpta Medica: Amsterdam, p. 87.

BEVERLEY, P., LINCH, D. & DELIA, D. (1980). Isolation of human haematopoietic progenitor cells using monoclonal antibodies. *Nature*, **287**, 332.

BHAN, A.K. & DES MARAIS, E.L. (1983). Immunohistologic characterization of major histocompatibility antigens and inflammatory cellular infiltrate in human breast cancer. *J. Natl Cancer Inst.*, **71**, 507.

BREARD, J. REINHERZ. E.L., KUNG, P.C., GOLDSTEIN, G. & SCHLOSSMAN, S.F. (1980). A monoclonal antibody reactive with human peripheral blood monocytes. *J. Immunol.*, **124**, 1943.

CALLARD, R.E., SMITH, C.M., WOMAN, C., LINCH, D., CAWLEY, J.C. & BEVERLEY, P.C.L. (1981). Unusual phenotype and function of an expanded subpopulation of T cells in patients with haematopoietic disorders. *Clin. Exp. Immunol.*, **43**, 497.

DAAR, A.S. & FABRE, J.W. (1983). The membrane antigens of human colorectal cancer cells: demonstration with monoclonal antibodies of heterogeneity within and between tumours and of anomalous expression of HLA-DR. *Eur. J. Cancer Clin. Oncol.*, **19**, 209.

DAAR, A.S., FUGGLE, S.V., TING, A. & FABRE, J.W. (1982). Anomalous expression of HLA-DR antigen on human colorectal cancer cells. *J. Immunol.*, **129**, 447.

DEKRESTER, T.A., CRUMPTON, M.J., BODMER, J.F. & BODMER, W.F. (1982). Two dimensional gel analysis of the polypeptides precipitated by a polymorphic HLA-DR1, 2, w6 monoclonal antibody: evidence for a third locus. *Eur. J. Immunol.*, **12**, 600.

DUKES, C.E. & BUSSEY, H.J.R. (1958). The spread of rectal cancer and its effect on prognosis. *Br. J. Cancer*, **12**, 309.

EREMIN, O., COOMBS, R.R.A. & ASHBY, J. (1981). Lymphocytes infiltrating human breast cancers lack K-cell activity and show levels of NK-cell activity. *Br. J. Cancer*, **44**, 166.

FLEMING, K.A., McMICHAEL, A., MORTON, J.A., WOODS, J. & McGEE, J.O.D. (1981). Distribution of HLA Class I antigens in normal human tissue and in mammary cancer. *J. Clin. Pathol.*, **34**, 779.

GUERRY, D., ALEXANDER, M.A., HERLYN, M.F. & 4 others. (1984). HLA-DR histocompatibility leukocyte antigens permit cultured human melanoma cells from early but not advanced disease to stimulate autologous lymphocytes. *J. Clin. Invst.*, **73**, 267.

HASKILL, J. (Ed). (1982). *Tumour Immunity in Prognosis: The Role of Mononuclear Cell Infiltration.* Marcel Dekker Inc., New York.

IOACHIM, H.L. (1976). The stromal reaction of tumours: an expression of immune surveillance. *J. Natl Cancer Inst.*, **57**, 465.

INTRONA, M., ALLEVENA, P., BIONDI, A., COLOMBO, N., VILLA, A. & MANTOVANI, A., (1983). Defective natural killer activity within human ovarian tumours: low numbers of morphologically defined effectors present *in situ*. *J. Natl Cancer Inst.*, **70**, 21.

KLARESKOG, L., FORSUM, U. & PETERSON, P.A. (1980). Hormonal regulation of expression of Ia-antigens on mammary gland epithelium. *Eur. J. Immunol.*, **101**, 958.

KUNG, P.C., GOLDSTEIN, G., REINHERZ, E.L. & SCHLOSSMAN, S.F. (1979). Monoclonal antibodies defining distinctive human T cell surface antigens. *Science*, **206**, 347.

LAMPERT, I.A., SUITTERS, A.J. & CHISHOLM, P.M. (1981). Expression of 1a antigen on epidermal keratinocytes in graft-versus-host disease. *Nature*, **293**, 149.

MASON, D.W., DALLMAN, M. & BARCLAY, A.N., (1981). Graft-versus-host disease induces expression of Ia antigens in rat epidermal cells and gut epithelium. *Nature*, **293**, 150.

McMICHAEL, A.J. (1978). HLA restriction of human cytotoxic lymphocytes specific for influenza virus associated with HLA-A2. *J. Exp. Med.*, **148**, 1458.

MOORE, M. (1984). Tumour resistance and the phenomenon of inflammatory-cell infiltration. In: *Handbook of Experimental Pharmacology* (eds. B.W. Fox & M. Fox) Springer-Verlag, Berlin, **72**, 143.

MOORE, M. & VOSE, B.M. (1981). Extravascular natural cytotoxicity in man: Anti-K562 activity of lymph node and tumour infiltrating lymphocytes. *Int. J. Cancer*, **27**, 265.

MURRAY, D., HOENO, A., DUTTON, J. & HAMPSON, L.G. (1975). Prognosis in colon cancer. A pathologic measurement. *Arch. Surg.*, **110**, 908.

NATALI, P.G., MARTINO, C.D., QUARANTA, V. & 4 others. (1981). Expression of Ia-like antigens in normal non-lymphoid tissues. *Transplantation*, **31**, 75.

ORTALDO, J.R., SHARROW, S.O., TIMONEN, T. & HERBERMAN, R.B. (1981). Determination of surface antigens on highly purified human NK cells by flow cytometry with monoclonal antibodies. *J. Immunol.*, **127**, 2401.

PERUSSIA, B., STARR, S., ABRAHAM, S., FANNING, V. & TRINCHIERI, G. (1983*a*). Human natural killer cells analysed by B73.1, a monoclonal antibody blocking Fc receptor functions. I. Characterisation of the lymphocyte subset reactive with B73.1. *J. Immunol.*, **130**, 2133.

PERUSSIA, B., ACUTA, O., TERHORST, C. & 4 others. (1983*b*). Human natural killer cells analysed by B73.1, a monoclonal antibody blocking Fc receptor functions. II. Studies of B73.1 antibody-antigen interaction on the lymphocyte membrane. *J. Immunol.*, **130**, 2142.

PIZZOLO, G., SEMENZATO, G., CHILOSI, M. & 5 others. (1984). Distribution and heterogeneity of cells detected by HNK-1 monoclonal antibody in blood and tissues in normal reactive and neoplastic conditions. *Clin. Exp. Immunol.*, **57**, 195.

REINHERZ, E.L., KUNG, P.C., GOLDSTEIN, G. & SCHLOSSMAN, W.F. (1979*a*). A monoclonal antibody with selective reactivity for functionally mature human thymocytes and all peripheral human T cells. *J. Immunol.*, **123**, 1312.

REINHERZ, E.L., KUNG, P.L., GOLDSTEIN, G. & SCHLOSSMAN, S.F. (1979*b*). Further characterisation of the human inducer T cell subset defined by monoclonal antibody. *J. Immunol.*, **123**, 2894.

REINHERZ, E.L., KUNG, P.C., GOLDSTEIN, G. & SCHLOSSMAN, S.F. (1980). A monoclonal antibody reactive with the human cytotoxic/suppressor T cell subset previously defined by a heteroantiserum termed TH2. *J. Immunol.*, **124**, 1301.

REINHERZ, E.L., MEUER, S.C. & SCHLOSSMAN, S.F. (1983). The delineation of antigen receptors on human T lymphocytes. *Immunol. Today*, **4**, 5.

ROGNUM, T.O., BRANDZAEG, P. & THORUD, E. (1983). Is heterogeneous expression of HLA-DR antigens & CEA along with DNA-profile variations evidence of phenotypic instability and clonal proliferation in human large bowel carcinomas? *Br. J. Cancer*, **48**, 543.

ROWE, D.J. & BEVERLEY, P.C.L. (1984). Characterisation of breast cancer infiltrates using monoclonal antibodies to human leucocyte antigents. *Br. J. Cancer*, **49**, 149.

RUITER, D.J., BHAN, A.K., HARRIST, T.J., SOHER, A.J. & MIHM., M.C. Jr. (1982). Major histocompatibility antigens and mononuclear inflammatory infiltrate in benign nevomelanocytic proliferations and malignant melonoma. *J. Immunol.*, **129**, 2808.

SELBY, W.S., JANOSSY, G., GOLDSTEIN, G. & JEWELL, D.P. (1981). T lymphocyte subsets in human intestinal mucosa; the distribution and relationship to MHC-derived antigens. *Clin. Exp. Immunol.*, **44**, 453.

SPRATT, J.S., & SPJUT, H.J. (1967). Prevalence and prognosis of individual clinical and pathologic variables associated with colorectal carcinoma. *Cancer*, **20**, 1976.

SVENNEVIG, J.L., LUNDE, O.C., HOLTER, J. & BJORGSVIK, D. (1984). Lymphoid infiltration and prognosis in colorectal carcinoma. *Br. J. Cancer*, **49**, 375.

THOMPSON, J. J., HERLYN, M.F., ELDER, D.E., CLARK, W.H., STEPLEWSKI, Z. & KOPROWSKI, H. (1982). Expression of DR antigens in freshly frozen human tumours. *Hybridoma*, **1**, 161.

TOTTERMAN, T.H., HAYRY, P., SAKSELA, E., TIMONEN, T., & EKLUND, B. (1988). Cytological and functional analysis of inflammatory infiltration in human malignant tumours. II. Functional investigations of the inflammatory cells. *Eur. J. Immunol.*, **8**, 872.

UCHIYAMA, T., NELSON, D.L., FLEISHER, T.A. & WALDMANN, T.A. (1981). A monoclonal antibody (anti-Tac) reactive with activated and functionally mature human T cells II. Expression of Tac antigen on activated cytotoxic killer T cells, suppressor cells and on one of two types of helper T cells. *J. Immunol.*, **126**, 1398.

UNDERWOOD, J.C.E. (1974). Lymphoreticular infiltration in human tumours: prognostic and biological implications: a review. *Br. J. Cancer*, **30**, 538.

VANKY, F., WILLEMS, J., KREICBERGS, A. & 6 others. (1983*a*). Correlation between lymphocyte-mediated auto-tumor reactivities and clinical course. I. Evaluation of 46 patients with sarcoma. *Cancer Immunol., Immunother.*, **16**, 11.

VANKY, F., PETERFFY, A., BOOK, K., WILLEMS, J., KLEIN, E. & KLEIN, G. (1983*b*). Correlation between lymphocyte-mediated auto-tumor reactivities and the clinical course. II Evaluation of 69 patients with lung carcinoma. *Cancer Immunol. Immunother.*, **16**, 17.

VOSE, B.M., GALLAGHER, P., MOORE, M. & SCHOFIELD, P.F. (1981). Specific and non-specific lymphocyte cytotoxicity in colon carcinoma. *Br. J. Cancer*, **44**, 846.

VOSE, B.M. & MOORE, M. (1979). Suppressor cell activity of lymphocytes infiltrating human lung and breast tumours. *Int. J. Cancer*, **24**, 579.

VOSE, B.M. & MOORE, M. (1984). Human tumour-infiltrating lymphocytes – a marker of host response. *Semin. Haematol.* (In press).

WATANABE, S., SATO, Y., KODAMA, T. & SHIMOSATO, Y. (1983). Immunohistochemical study with monoclonal antibodies on immune response in human lung cancers. *Cancer Res.* **43**, 5883.

WATT, A.G. & HOUSE, A.K. (1978). Colonic carcinoma. A quantitative assessment of lymphocyte infiltration at the periphery of colonic tumours related to prognosis. *Cancer*, **41**, 279.

WERKMEISTER, J.A., PIHL, E., NIND, A.A.P., FLANNERY, G.R. & NAIRN, R.C. (1979). Immunoreactivity by intrinsic lymphoid cells in colorectal carcinoma. *Br. J. Cancer*, **40**, 839.

WHITWELL, H.L., HUGHES, H.P.A., MOORE, M. & AHMED, A (1984). Expression of major histocompatibility antigens and leucocyte infiltration in benign and malignant human breast disease. *Br. J. Cancer*, **49**, 161.